# Vaccination gaps in decentralized emergency response: understanding immunization barriers among volunteer firefighters. A cross-sectional mixed methods study

**DOI:** 10.3389/fpubh.2026.1803949

**Published:** 2026-04-10

**Authors:** Andrea Baron, Laura Over-Müller, Swantje Brandt, Ole Franzen, Ingo Meßer, Kai Mortensen, Peer Franzen, Peter Münstedt, Bernhard Elsner, Maik von der Forst, Markus Ries, Daniel Drömann, Patrick Ristau, Klaas F. Franzen

**Affiliations:** 1 Department of Anaesthesiology and Intensive Care Medicine, University Hospital Schleswig-Holstein, Luebeck, Germany; 2 Medical Clinic III, University of Luebeck, Luebeck, Germany; 3 Ministry of the Interior, Municipal Affairs, Housing and Sports, Kiel, Germany; 4 Stormarn Technical Operations Management, Stormarn District Fire Brigade Association, Travenbrück, Germany; 5 Reinfeld Volunteer Fire Brigade, Reinfeld, Germany; 6 Asklepios Klinik am Kurpark, Bad Schwartau, Germany; 7 Cardiology Kiel, Kiel, Germany; 8 Hamburg Fire Brigade, Ministry of the Interior and Sport of the Free and Hanseatic City of Hamburg, Hamburg, Germany; 9 Anaesthesiology and Intensive Care Medicine, DRK Hospital Ratzeburg, Ratzeburg, Germany; 10 Institute für Gesundheitswissenschaften, University of Luebeck, Luebeck, Germany; 11 Crisis and Disaster Management Unit, Medical Faculty Heidelberg, Heidelberg University, Heidelberg, Germany; 12 Airway Research Center North, Member of the German Center for Lung Research (DZL), Großhansdorf, Germany; 13 Faculty of Applied Health Sciences, Deggendorf Institute of Technology, Deggendorf, Germany; 14 Institute for Social Medicine and Epidemiology, University of Lübeck, Luebeck, Germany

**Keywords:** communicable diseases, emergency response, health promotion, resilience, vaccination, volunteering

## Abstract

**Introduction:**

Volunteer firefighters are crucial for decentralized emergency response systems but operate outside standardized occupational health frameworks. Despite elevated exposure to biological hazards, systematic data on vaccination knowledge, immunization status, and preventive health engagement in this population are limited. To better understand the determinants of their preventive health engagement, and to inform strategies that strengthen operational readiness and public health resilience, this study examined vaccination behavior among German volunteer firefighters and identified determinants of immunization uptake.

**Methods:**

Between April 2025 and August 2025, semi-structured interviews were conducted with 150 active volunteer firefighters. Data were analyzed using inductive qualitative content analysis, following Mayring's framework. Vaccination status, evaluation of vaccination, and willingness to undergo medical surveillance were assessed. K-means cluster analysis identified behavioral typologies, and Spearman's correlations examined the associations between risk perception, social integration, and vaccination behavior.

**Results:**

Approximately one-third of the participants could not confirm their current immunization status. The most common vaccinations recorded among the 150 firefighters were tetanus (*n* = 110) and hepatitis B (*n* = 100). Three distinct behavioral clusters emerged: health-engaged prevention-oriented individuals (predominantly healthcare workers), vaccine-supportive but surveillance-resistant firefighters (craftsmen/trading backgrounds), and vaccine-ambivalent personnel open to alternative prevention (logistics/technical fields). Hazard perception was weakly correlated with vaccination status (ρ = +0.300, *p* = 0.035). Social proximity correlated positively with vaccination willingness (ρ = +0.33, *p* = 0.037) and negatively with hazard perception (ρ = −0.43, *p* = 0.024). Vaccination rates varied by occupation, ranging from 100% in IT/technical professionals, to 66.7% in healthcare workers, and 50 % craftsmen/trading workers.

**Discussion:**

Substantial vaccination knowledge gaps, incomplete documentation, and heterogeneous behavioral patterns shaped by professional background and institutional trust characterized the population surveyed. Effective interventions require differentiated strategies that address structural barriers, transparent communication, integration with valued occupational health surveillance, and recognition of distinct motivational profiles across volunteer firefighter subgroups.

## 1 Introduction

Volunteer firefighters are crucial components of decentralized emergency response systems. Operating outside standardized occupational health schemes, they face structural gaps in access to vaccinations, medical surveillance, and institutional risk communication (1–3). Despite elevated exposure to biological hazards, non-professional emergency personnel remain less consistently integrated into preventive health frameworks than their professional counterparts (4, 5). This disparity becomes particularly evident in immunization, where adult and occupational vaccines receive substantially less emphasis than childhood immunizations in emergency service schemes (6, 7).

Documentation of vaccination status among volunteer emergency personnel is challenging. German population data shows that only 36% of adults possess complete vaccination records, with 40% reporting uncertain status due to paper-based systems and absent centralized registries (8). This documentation deficit is amplified in volunteer emergency services, where self-reporting limitations and incomplete record-keeping create uncertainty regarding immunization coverage (8, 9). International vaccination regulations for emergency services vary widely, with most jurisdictions providing recommendations rather than mandates (10, 11), disproportionately affecting volunteer units lacking dedicated occupational health infrastructure (12).

Research demonstrates that vaccination knowledge alone is insufficient to drive immunization uptake among emergency personnel (13, 14). Instead, attitudes toward vaccine safety, risk perception, institutional trust, professional background, and social norms exert stronger influences on vaccination behavior (13–15). Professional firefighters embedded in structured organizations demonstrate substantially higher vaccination rates than volunteers, whose attitudes are more heavily shaped by personal beliefs and social networks (16). Paradoxically, while perceived infection risk increases vaccination acceptance (17, 18), social proximity to fellow firefighters correlates positively with vaccination willingness but negatively with hazard perception, suggesting normative rather than risk-based pathways to immunization uptake (16, 19).

Institutional trust emerges as a critical determinant of vaccination behavior in volunteer organizations (19, 20). Psychological and attitudinal factors, conceptualized within Health Belief Model frameworks emphasizing perceived susceptibility, severity, benefits, and barriers, often surpass demographic predictors in explaining vaccination intention (21, 22). Barriers extend beyond knowledge deficits to encompass safety concerns, communication gaps, and previous adverse experiences (23, 24). Transparent risk-benefit communication is essential for maintaining trust and preventing vaccine refusal cascades in close-knit volunteer organizations (25). Medical screening programs integrating vaccination into routine occupational health assessments demonstrate modest but meaningful increases in immunization acceptance (5, 26).

Despite recognized importance for operational capability (9, 27), vaccination remains poorly integrated into operational safety frameworks among firefighters (5), and systematic vaccination monitoring systems are largely absent in volunteer services (8, 9). The preventive health engagement of volunteer firefighters remains insufficiently explored, with prior research predominantly examining healthcare workers and professional emergency services (24, 28), while rarely addressing non-professional first responders or considering the implications of incomplete vaccination coverage for systemic risk and operational resilience.

This study examined vaccination knowledge, willingness, and immunization status among active volunteer firefighters in Germany, with a focus on organizational structures, social determinants, and perceived barriers to preventive engagement. Two research questions guided the investigation:

(1) *What is the current level of vaccination-related knowledge, willingness, and coverage among volunteer firefighters?*
(2) *How do individual and organizational factors (including risk perception, professional background, and perceived institutional responsibility) predict preventive health behavior, including participation in vaccination and medical surveillance?*

By integrating qualitative insights and quantitative analytical approaches, this study aimed to inform public health interventions, contribute to occupational vaccination theory, and support scalable frameworks for improving health resilience in volunteer-based civil protection systems.

## 2 Methods

### 2.1 Study design and reporting standards

This investigation employed a qualitative exploratory design to examine the vaccination attitudes, immunization behaviors, and preventive health practices of volunteer firefighters in Germany. This study was reported in accordance with the Standards for Reporting Qualitative Research (SRQR) guidelines (29). This research was conceived as part of a larger two-phase investigation on occupational health and safety among volunteer fire services, utilizing the identical methodological foundations as previously described for hazard perception assessment. In this context, participants provided self-assessed hazard perception ratings on a numerical scale ranging from 1 (very low perceived danger) to 10 (very high perceived danger), derived from interview responses regarding their subjective risk appraisal across deployment scenarios. These scores were referenced in the present analysis to contextualize behavioral cluster characteristics. The current analysis specifically focuses on thematic domains related to vaccination status, infection prevention knowledge, and engagement with medical surveillance programs.

A qualitative approach was adopted to capture the nuanced perspectives, beliefs, and decision-making processes surrounding immunization practices within the group of volunteer firefighters. Semi-structured interviews enabled the participants to articulate their experiences with vaccination requirements, understanding of infection risks, and expectations regarding occupational health support.

### 2.2 Research team and reflexivity

This study was conducted by an interdisciplinary research team with expertise in occupational medicine, public health, qualitative methodologies, and emergency services. The team members possessed extensive experience in conducting semi-structured interviews and applying inductive qualitative content analysis across diverse healthcare contexts. The lead investigators included specialists in infection prevention and occupational health surveillance, ensuring domain-specific competence in interpreting vaccination-related narratives and preventive health behaviors. Regular research group meetings were held throughout data collection and analysis to maintain methodological rigor and reflexive awareness of potential biases.

### 2.3 Participant recruitment and selection

Between April 2025 and August 2025, 150 active volunteer firefighters in Germany were recruited for individual interviews. Participant recruitment employed a multichannel strategy encompassing direct contact through municipal and regional fire brigade networks, targeted announcements in firefighter-specific social media groups and online forums, and snowball sampling techniques facilitated by initial participants. This approach ensured broad geographic and demographic representation across Germany.

The eligibility criteria were as follows: (a) current active membership in a volunteer fire service in Northern Germany, (b) participation in at least one operation within the preceding 12 months, and (c) willingness and capacity to engage in an interview lasting up to 60 min. Participants represented diverse demographic profiles, operational experience levels, professional backgrounds, and geographic contexts (urban centers, rural communities, and suburban areas), thereby enabling a comparative analysis across multiple dimensions relevant to vaccination uptake and preventive health engagement.

### 2.4 Sampling strategy and sample characteristics

Purposive sampling was employed to ensure heterogeneity across key variables, including age, gender, years of service, operational role, and professional occupation. The sampling framework intentionally included firefighters from both healthcare and non-healthcare professional backgrounds to explore potential differences in vaccination knowledge and behavior among them. Geographic diversity was prioritized to capture variations in regional health infrastructure and organizational vaccination policies. The final sample achieved broad representational coverage across these domains, facilitating the examination of subgroup-specific patterns in immunization attitudes and preventive health participation.

### 2.5 Data collection instruments

A semi-structured interview guide was iteratively developed through consultations with subject matter experts in occupational health, infectious disease prevention, and fire service operations. The guide was refined following pilot interviews (*n* = 5) with volunteer firefighters who were not included in the main sample. These preliminary conversations informed the formulation of questions, sequence optimization, and probe development. Interviews were conducted in German language.

The final interview guide (Supplementary Appendix 1) addressed the following thematic domains:

- General preventive measures employed at operational scenes
- Personal vaccination status and awareness of immunization records
- Knowledge regarding vaccine-preventable diseases relevant to emergency response
- Perceived utility and relevance of specific vaccinations for firefighting activities
- Information needs and preferences regarding vaccination decision-making
- Attitudes toward incentives for vaccination uptake
- Awareness of operational readiness implications related to incomplete immunization
- Willingness to participate in occupational medical surveillance programs
- Expectations regarding medical screening content and format
- Preferences concerning health documentation systems (e.g., digital health cards)

The semi-structured format allowed interviewers to pursue emergent themes and participant-initiated topics, while maintaining consistency in core content coverage across interviews.

### 2.6 Interview procedures

All interviews were conducted by trained research team members. Interviews were conducted either face-to-face at fire stations, community facilities, or public venues convenient to the participants, or remotely via secure videoconferencing platforms (Cisco Webex; Cisco Systems; San José, USA), depending on the participant's preference and geographic considerations (30). Remote interviewing enabled the inclusion of participants from geographically dispersed locations while maintaining data quality.

Each interview session began with an explanation of the purpose of the research, voluntary participation, confidentiality protection, and consent procedures. Following written informed consent, the interviews proceeded according to a semi-structured guide, with durations ranging from 35 to 75 min (mean = 52 min). All interviews were digitally audio-recorded with explicit participant permission. The interviewers maintained field notes documenting contextual observations, non-verbal communication, and reflexive impressions to supplement the audio data.

### 2.7 Data management and transcription

Audio recordings were transcribed verbatim using the f4transkript software (Dr. Dresing & Pehl GmbH, Marburg, Germany), adhering to the standardized transcription conventions. The transcripts were verified by comparing the transcribed text with the original recordings. Participant identifiers were systematically replaced with alphanumeric pseudonyms during transcription to ensure anonymization of the data. All data files, including audio recordings, transcripts, and field notes, were stored on password-protected, encrypted servers maintained by the university's information technology infrastructure in full compliance with the General Data Protection Regulation (GDPR) requirements. Following the study's completion, the linkage files connecting pseudonyms to participant identities were permanently destroyed, rendering the dataset fully anonymized.

### 2.8 Analytical approach

A mixed methods approach was chosen to combine in-depth qualitative insights into firefighters' perceptions and decision-making with quantitative pattern identification, allowing for a more comprehensive understanding of the factors shaping vaccination behavior.

Specifically, interview transcripts were analyzed using inductive qualitative content analysis, following Mayring's methodological framework (31). This approach emphasizes systematic, rule-guided text reduction while preserving the essential content meaning through iterative categorization and abstraction (32).

The analytical process proceeded through the following phases:

1. *Familiarization and Initial Coding:* All transcripts were thoroughly read by multiple team members to achieve immersion in the data. Initial open coding identified meaningful text segments related to vaccination attitudes, preventive behaviors, and health surveillance engagement. Codes were developed inductively from the data rather than imposed a priori.
2. *Category Development:* Coded segments were systematically compared and grouped into provisional subcategories representing the shared thematic content. Subcategories were subsequently abstracted into higher-order main categories, capturing the overarching dimensions of vaccination behavior and prevention practices.
3. *Inter-Rater Reliability Assessment:* To ensure analytical consistency and category validity, two independent coders analyzed a subset of 30 transcripts (20% of the total sample) using the preliminary coding framework. Coding discrepancies were identified and resolved through a structured consensus. The coding scheme was iteratively refined based on these deliberations.
4. *Systematic Application:* The final category system was systematically applied to the complete dataset using MAXQDA Analytics Pro 2020 (VERBI Software GmbH, Berlin, Germany). This software facilitated systematic code assignment, retrieval of coded segments, and exploration of patterns across participant subgroups.
5. *Pattern Identification and Interpretation:* Following complete coding, the research team conducted comparative analyses to identify recurring patterns, convergent and divergent perspectives, and subgroup-specific variations in vaccination attitudes and preventive health behaviors. Descriptive quantification of category frequencies was employed where appropriate to illuminate the distribution of perspectives sample while maintaining a focus on the interpretive depth characteristic of within the qualitative inquiry.

### 2.9 Subgroup analyses

Although this study employed a qualitative methodology, the substantial sample size (*n* = 150) afforded opportunities for a descriptive comparative analysis across participant subgroups. Coded data were examined for patterns associated with demographic characteristics (age and sex), operational variables (years of service and deployment frequency), professional background (healthcare vs. non-healthcare occupations), and geographic context (urban vs. rural fire services). Professional occupations were assessed through the open-ended interview question “What is your main occupation?” Free-text responses were coded inductively and *post hoc* into seven occupational categories (IT and technical professionals, social and educational workers, healthcare professionals, blue-collar/emergency services workers, office/administrative staff, craftsmen/trading workers, and other/miscellaneous) by the research team through consensus-based classification. Given the predominantly unambiguous nature of the reported occupations (e.g., nurse, electrician, IT specialist), category assignment was straightforward in the vast majority cases. These comparisons enriched our understanding of the factors that potentially influence vaccination uptake and engagement with preventive health initiatives.

### 2.10 Cluster analysis of vaccination patterns

To identify distinct profiles of vaccination-related attitudes and behaviors, an exploratory cluster analysis was conducted using coded categorical data derived from the qualitative interviews. Participants were characterized across three dimensions extracted from the thematic analysis with each theme assigned a binary attribution property for each individual: (1) current vaccination status (adequate [1] vs. inadequate [0], (2) evaluation of vaccination utility (positive [1] vs. negative/ambivalent [0]), and (3) willingness to participate in medical surveillance (high [1] vs. low 0). Cluster-level values reported in the results represent the proportion of individuals within each cluster meeting the positive criterion (i.e., 1.0 indicates all members positive, and 0.0 indicates all members negative). A k-means clustering algorithm was applied to identify internally homogeneous participant groups with maximally distinct characteristics across these dimensions. The optimal number of clusters was determined using silhouette coefficient analyses. This supplementary quantitative exploration of qualitative data enabled the identification of coherent subpopulations with differing vaccination profiles, informing targeted intervention development.

### 2.11 Methodological rigor and trustworthiness

Multiple strategies were employed to enhance the trustworthiness and credibility of the findings.

- Investigator triangulation: Multiple researchers independently coded and analyzed the data, with regular consensus meetings to reconcile interpretations.
- Maximum variation sampling: Deliberate recruitment across diverse demographic, geographic, and professional characteristics enhanced transferability.
- Audit trail: Comprehensive documentation of analytical decisions, coding evolution, and category development was maintained throughout the study.
- Member checking: Selected participants (*n* = 12) were invited to review the preliminary findings to assess resonance with their experiences.

### 2.12 Reporting and synthesis

The findings are presented thematically and organized according to the main categories identified through inductive content analysis. Representative quotations are included to illustrate key themes and provide a transparent linkage between the interpretations and primary data. Participant quotations are identified by pseudonym codes to maintain confidentiality while enabling readers to trace perspectives across themes. Where quantitative descriptors (e.g., “most participants,” “a minority”) are used, these reflect systematic frequency assessments within the coded dataset while preserving the interpretive orientation of the qualitative analysis.

### 2.13 Ethical considerations

This study was approved by the Ethics Committee of the University of Lübeck, Germany, (reference number AZ 2023-123; final approval 29.04.2025). All participants provided written informed consent after receiving comprehensive information regarding the study aims, procedures, voluntary participation, benefits and risks, confidentiality safeguards, and the right to withdraw without consequences. Participants were assured that their responses would be anonymized and that participation or non-participation would not influence their standing within the fire service organizations. No compensation was provided for participation in the study. Data handling procedures strictly adhered to the GDPR requirements and institutional data protection policies.

## 3 Results

### 3.1 Vaccination status and knowledge

**Figure 1:**
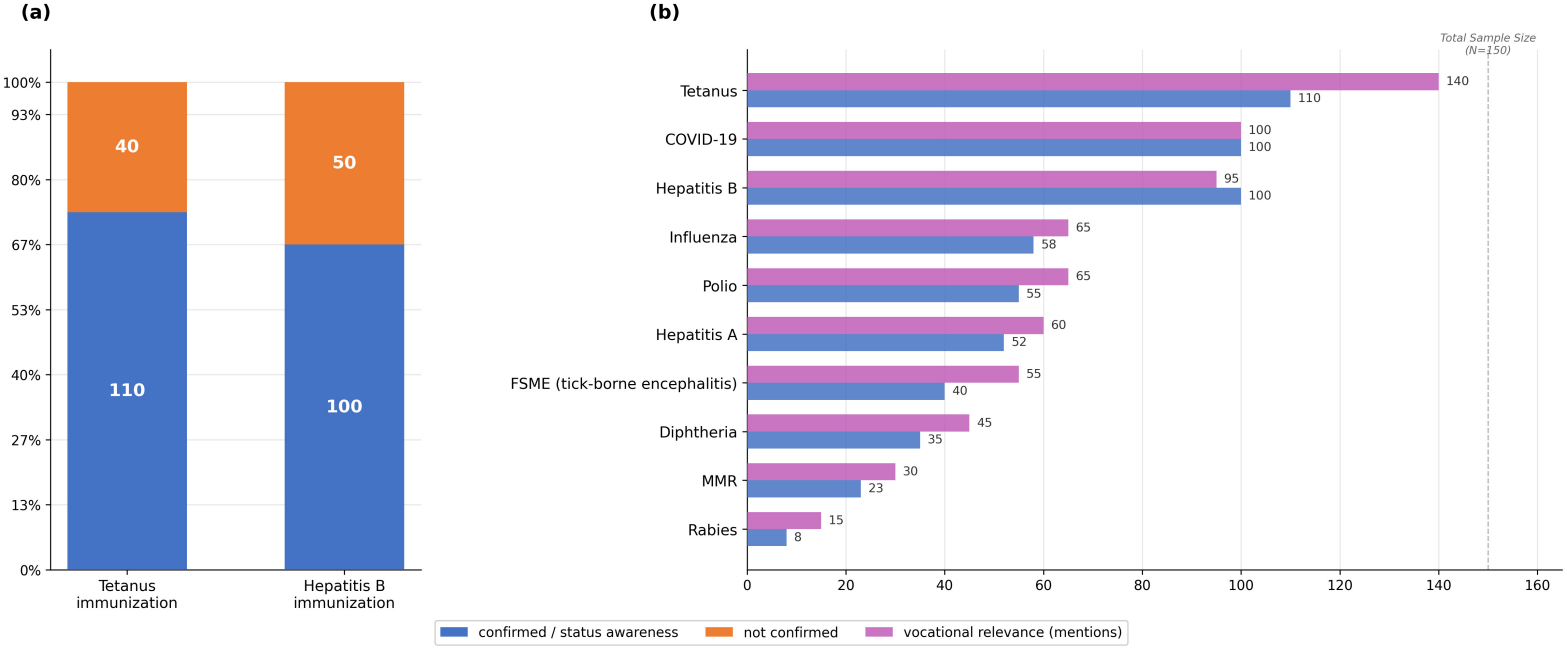
Firefighters' vaccination status and knowledge regarding vaccinations. **(a)** Firefighters' awareness of vaccination status for tetanus and hepatitis B; **(b)** side-by-side comparison of individual vaccination status awareness (confirmed) and vocational relevance awareness (mentions) per vaccine across the sample (*N* = 150).

Among the 150 participants (Table 1), awareness of the current immunization status varied considerably. Approximately one-third of the participants were definitively unable to confirm their vaccination status for core occupational immunizations. Tetanus was the most frequently acknowledged vaccination (*n* = 110 confirmed recipients), followed by hepatitis B (*n* = 100). Knowledge of MMR, polio, diphtheria, and FSME (Frühsommer-Meningoenzephalitis; tick-borne encephalitis) remained markedly lower. When asked to identify occupationally relevant vaccinations, tetanus (140 mentions) was nearly universally mentioned, followed by COVID-19 (*n* = 100), hepatitis B (*n* = 95), polio (*n* = 65), influenza (*n* = 65), hepatitis A (*n* = 60), and FSME (n = 55). Diphtheria (*n* = 45), measles, mumps, and rubella (MMR; *n* = 30), and rabies (*n* = 15) received progressively fewer mentions, reflecting limited awareness beyond the most emphasized immunizations. Figure 1 illustrates the distribution of vaccination status awareness and mentions of vocationally relevant vaccinations across the sample.

**Table 1:** Participant characteristics (*N* = 150).

Characteristic	*n* or value	% or range
Demographics		
Age, years		
Mean (SD)	40.6 (14.5)	
Range		18–65
Sex		
Male	137	91.3
Female	13	8.7
Fire service experience		
Entry year (mean)	2,013.6	
Period of entry		
2000–2009	34	22.7
2010–2019	99	66.0
2020–2024	17	11.3
Occupational background		
Technical trades & crafts	26	17.3
Healthcare	21	14.0
Administration & office	14	9.3
Protection & rescue services	9	6.0
Education & social services	8	5.3
Logistics & transport	6	4.0
IT & technology	2	1.3
Other/not specified	64	42.7
Educational level		
Vocational training^a^	23	15.3
Advanced training/master craftsman	18	12.0
No formal training/semi-skilled	15	10.0
University degree	7	4.7
Not specified/unclear	87	58.0

SD, Standard deviation.

^a^Educational level was inferred from reported occupation where explicit information was not provided.

### 3.2 Infection risk perception

The participants demonstrated a nuanced, context-specific awareness of infectious hazards in operational activities. As such, viral infections represented the predominant concern (*n* = 350 mentions across contexts), substantially exceeding references to bacterial infections (*n* = 125). Among specific pathogens, tetanus (*n* = 75), FSME (*n* = 70), and influenza (*n* = 70) were most frequently identified, followed by HIV (*n* = 65), hepatitis C (*n* = 65), hepatitis B (*n* = 60), MRSA (methicillin-resistant Staphylococcus aureus) (*n* = 60), tuberculosis (*n* = 55), COVID-19 (*n* = 50), and norovirus (*n* = 50). This distribution indicates an understanding that extends beyond vaccine-preventable diseases to antimicrobial-resistant organisms and fluid-borne pathogens that require behavioral and situational protective measures.

### 3.3 Vaccination utility and benefit recognition

Most participants understood vaccination primarily as a means of providing individual protection, with substantially fewer demonstrating an awareness of herd immunity principles. Knowledge of population-level immunity was concentrated among healthcare professionals, suggesting that this concept requires more specialized training. Professional background strongly shaped the overall depth of vaccination knowledge, with healthcare workers exhibiting the highest level of immunological literacy.

### 3.4 Occupational health surveillance attitudes

Most participants were willing to participate in medical surveillance programs, although this was accompanied by substantial privacy, autonomy, and operational restriction concerns. Participants emphasized that surveillance acceptance requires robust confidentiality safeguards, voluntary participation frameworks, convenient accessibility, and explicit separation between health monitoring activities and decisions about deployment eligibility.

Expectations for surveillance content were comprehensive and multidimensional in nature. Cardiovascular assessment (blood pressure, ECG, and cardiac risk stratification) was most frequently prioritized, followed by respiratory function testing, cancer screening, comprehensive blood chemistry panels, vaccination titer assessment, musculoskeletal examinations, and vision and hearing evaluations. Participants also emphasized the importance of toxicology and biomarker testing for occupational exposures, as well as targeted infectious disease screening. Beyond physical examinations, participants expressed interest in receiving health education and preventive guidance, including nutrition counseling, exercise and fitness recommendations tailored to firefighting demands, injury prevention, and sleep or fatigue management. Mental health screening was viewed as potentially valuable, but acceptable only under strict confidentiality with assurances that participation would not result in operational consequences.

### 3.5 Information needs and incentive preferences

Participants identified several key information needs prior to vaccination, including concise explanations of disease risks and vaccine mechanisms, accessible and intuitive written materials for self-paced review, and transparent disclosure of potential adverse effects, covering both common reactions and the frequency of serious events. They also emphasized the importance of operationally relevant risk-benefit information, and guidance on post-vaccination readiness for deployment.

Regarding strategies to enhance vaccination uptake, participants expressed comparable levels of support for symbolic recognition (certificates, badges, and institutional acknowledgment) and material incentives, such as financial compensation or equipment upgrades. However, many participants rejected external inducements altogether, stressing the importance of autonomous, evidence-based decision-making. Several participants argued that reducing structural barriers, such as cost, access, and time constraints, would be more effective than providing positive incentives. A minority opposed any kind of incentive, viewing them as potentially manipulative or indicative of institutional distrust.

### 3.6 Awareness of vaccination-based operational requirements

When informed that incomplete immunization could lead to deployment restrictions, most participants expressed willingness to comply with vaccination requirements when framed as occupational health prerequisites comparable to other safety standards. This acceptance was especially pronounced among healthcare professionals and individuals with military experience, for whom immunization mandates were already routine. However, a minority voiced principled objections, perceiving such requirements as coercive or infringing on personal autonomy.

### 3.7 Digital health documentation

A strong majority supported the use of digital health information cards containing vaccination status, blood type, medications, allergies, and organ donor designations. Participants cited improved emergency care quality and consolidated record-keeping as key advantages. However, acceptance was contingent on robust data protection safeguards, user control over information sharing, voluntary participation, and transparent governance mechanisms that prevent unauthorized access or discriminatory use.

### 3.8 Cluster analysis: vaccination behavior profiles

K-means cluster analysis of vaccination status, vaccination evaluation, and surveillance willingness identified three distinct behavioral profiles.

Cluster 0 (Vaccination-Ambivalent, Surveillance-Open): Participants showed low vaccination evaluation (0.0), moderate vaccination status (0.40), and high willingness to participate in surveillance (0.80). The mean age was 36.1 years, and this group exhibited the lowest hazard perception score (5.9). Members were predominantly employed in logistics and technical professions. This profile suggests younger firefighters who lack strong vaccination convictions but remain generally open to health engagement.

Cluster 1 (Health-Engaged, Prevention-Oriented): Participants demonstrated high vaccination evaluation (1.0), moderate-to-complete vaccination status (0.41–1.0), and high surveillance willingness (1.0). Their mean age was 38.2 years, with a moderate hazard perception score (6.1). The cluster consisted mainly of healthcare and social service professionals and represented individuals who were broadly health-engaged and supportive of preventive measures.

Cluster 2 (Vaccination-Supportive, Surveillance-Resistant): Participants displayed high vaccination evaluation (1.0), moderate vaccination status (0.50), but no willingness to participate in surveillance (0.0). The mean age was 40.5 years, and they had the highest hazard perception score (6.4). This group, largely composed of craft and trade workers, illustrates a paradox in which experienced firefighters value vaccination pragmatically but resist broader medical monitoring, potentially reflecting institutional distrust or concerns about operational restrictions.

An extended cluster analysis incorporating hazard perception further refined these profiles: Cluster 0e showed a medium vaccination status (0.5), positive vaccination evaluation (1.0), no surveillance willingness (0.0), high hazard perception (7.2), and was predominantly male (31 men, 3 women) from craft and logistics backgrounds.

Cluster 1e demonstrated complete vaccination (1.0), positive vaccination evaluation (1.0), high surveillance willingness (1.0), medium hazard perception (5.8), and consisted of healthcare and social service professionals (*n* = 12).

Cluster 2e was characterized by no vaccination (0.0), negative vaccination evaluation (0.0), low surveillance willingness (0.3), high hazard perception (6.7) and was composed mainly of IT and technical professionals [8 men, 2 women)]. This refined clustering revealed a subgroup of vaccine-refusing individuals with elevated hazard awareness, suggesting principled opposition rather than a lack of knowledge.

### 3.9 Correlational patterns and demographic associations

Spearman's correlation analysis showed that age was not significantly associated with vaccination status (ρ = −0.086, *p* = 0.434). Hazard perception demonstrated a weak positive correlation with vaccination status (ρ = +0.300, *p* = 0.035), indicating that firefighters with higher risk awareness maintained better immunization compliance. Social proximity to fellow firefighters correlated positively with vaccination willingness (ρ = +0.33, *p* = 0.037) but negatively with hazard perception (ρ = −0.43, *p* = 0.024), suggesting that socially integrated members perceive lower threat levels, yet exhibit higher vaccination acceptance through normative influences. Hazard perception and vaccination willingness were also weakly positively associated (ρ = +0.26, *p* = 0.045).

### 3.10 Professional background and vaccination patterns

Vaccination rates varied systematically across occupational categories: IT and technical professionals (100% vaccinated), social and educational workers (70.6%), healthcare professionals (66.7%), blue-collar/emergency services workers (66.7%), office/administrative staff (66.7%), other/miscellaneous (61.1%), and craftsmen/trading workers (50%). Healthcare and social service professionals demonstrated the highest baseline uptake, whereas craft and trade workers showed the most even distribution between fully vaccinated and incompletely vaccinated individuals.

Logistic regression examining predictors of high hazard perception identified occupational field (logistics/transport) as marginally significant (*p* = 0.047), with no significant effects for gender, year of entry year, or deployment frequency. Professional background and entry motivation were systematically associated (χ^2^ = 33.45, *df* = 28, *p* = 0.009), with technical occupations showing weak significance (*B* = 1.64, *p* = 0.046).

### 3.11 Preventive practices at operational scenes

The utilization of personal protective equipment (PPE) was universally identified as a foundational infection prevention strategy. Respiratory protection (self-contained breathing apparatus (SCBA), N95 respirators, and surgical masks), gloves used for medical responses, and eye protection (inconsistently applied) constituted the primary barriers. Hand hygiene practices varied substantially, with some participants reporting rigorous protocols while others noted delays due to shortfalls in field logistics. Equipment decontamination procedures showed considerable interdepartmental variation. Regular training, adherence to procedural protocols (scene size-up and hazard assessment), and specialized infection control education were recognized as systemic prevention strategies. Notably, vaccination was seldom mentioned spontaneously within general preventive measures, suggesting limited cultural integration of immunization into operational safety frameworks.

## 4 Discussion

This study fills a critical research gap by providing the first comprehensive examination of vaccination behavior among volunteer firefighters in Germany, revealing previously undocumented structural, social, and behavioral determinants of immunization in a decentralized emergency response system. Specifically, this work reveals substantial heterogeneity in vaccination knowledge, attitudes, and behaviors in this population, with implications for occupational health policy and disaster preparedness. Three principal findings emerged: substantial knowledge gaps regarding occupational immunization recommendations, systematic variation in vaccination uptake as a function of professional background, and a weak association between risk perception and preventive behavior. These patterns are consistent with established theoretical frameworks and highlight barriers specific to decentralized emergency response systems.

### 4.1 Vaccination knowledge and documentation deficits

Approximately one-third of the participants could not confirm their current immunization status, corroborating prior findings that volunteer emergency responders lack reliable vaccination documentation (6). This aligns with recent German population data demonstrating that only 36% of adults possess complete vaccination records, with 40% reporting an uncertain status due to paper-based systems and the absence of centralized registries (8). Among our cohort, tetanus awareness substantially exceeded knowledge of hepatitis B, FSME, and other occupationally relevant immunizations—a pattern consistent with international studies showing that adult and occupational vaccine guidance disproportionately emphasizes childhood immunizations while neglecting first responder-specific risks (7, 28).

The dominance of tetanus recognition likely reflects the cultural salience of injury-associated infections rather than systematic occupational health education. This knowledge stratification creates vulnerability: while participants demonstrated sophisticated awareness of diverse pathogens in operational scenes (viral infections *n* = 350 mentions, bacterial *n* = 125), this contextual understanding did not translate into comprehensive immunization uptake. The disconnect between infection risk awareness and vaccination behavior suggests that knowledge alone is insufficient to drive preventive action (14, 15), a finding consistent with Health Belief Model predictions that perceived susceptibility and severity must align with actionable pathways and institutional support (22).

### 4.2 Professional background as vaccination determinant

Vaccination rates varied systematically by occupation, with healthcare professionals (66.7%), social service workers (70.6%), and IT/technical professionals (100%, though with a small sample size) demonstrating higher uptake than craft/trade workers (50%). This stratification parallels the findings from professional emergency services, showing that workplace norms, organizational structure, and role-specific health requirements strongly influence vaccination attitudes (16). Healthcare professionals benefit from mandatory immunization policies, routine occupational health integration, and peer cultures emphasizing evidence-based prevention, advantages largely absent in volunteer fire services, where engagement is altruistic and institutional health infrastructure is minimal.

The paradoxical finding that some clusters demonstrated high vaccination evaluation but low surveillance participation (Cluster 2: craft/trade backgrounds, 40.5 years mean age) suggests that experienced firefighters may view vaccination pragmatically for acute injury protection while resisting medical monitoring perceived as threatening operational autonomy. This mirrors research on institutional trust in volunteer organizations, where perceived institutional overreach can undermine cooperation despite general health consciousness (19).

### 4.3 Risk perception and vaccination behavior

Hazard perception demonstrated a weak positive correlation with vaccination status (ρ = +0.300, *p* = 0.035), indicating that while risk awareness contributes to immunization decisions, it is not determinative. This modest association aligns with prior literature, showing that perceived infection risk increases vaccination acceptance among firefighters; however, complacency and underestimation of the threat remain significant barriers (17, 18). Notably, social proximity was positively correlated with vaccination willingness (ρ = +0.33, *p* = 0.037), despite its negative association with hazard perception (ρ = −0.43, *p* = 0.024)—suggesting that vaccination uptake among socially integrated firefighters operates through normative compliance and institutional trust rather than personal risk assessment (19, 20).

The three-cluster typology identified distinct behavioral profiles: health-engaged prevention-oriented individuals (predominantly healthcare workers), vaccine-supportive but surveillance-resistant experienced firefighters (craft/trades), and vaccine-ambivalent younger personnel open to alternative prevention (logistics/technical). This heterogeneity necessitates differentiated intervention strategies rather than uniform campaigns, recognizing that barriers vary across subpopulations (21).

### 4.4 Barriers to vaccination and surveillance engagement

Participants emphasized information transparency, adverse effect disclosure, and operational readiness concerns as critical pre-vaccination priorities, echoing evidence that healthcare workers' vaccine hesitancy stems primarily from safety concerns and communication deficits rather than anti-vaccine ideology (23, 24). Previous adverse experiences powerfully shape future vaccination decisions (25); thus, transparent risk-benefit communication is essential for maintaining trust and preventing vaccine refusal cascades within close-knit volunteer organizations.

Regarding incentives, symbolic recognition and material rewards received comparable endorsements, although substantial segments rejected external inducements as undermining autonomous decision-making. This ambivalence reflects broader debates about vaccination incentive ethics (33, 34): while modest financial incentives demonstrably increase COVID-19 uptake (35), they risk signaling institutional distrust in vaccine value and may alienate individuals motivated by intrinsic health values rather than extrinsic rewards.

Strong support for occupational health surveillance (majority willingness) contingent on confidentiality protections and operational non-consequences suggests that volunteer firefighters would engage in comprehensive preventive programming if it is structured appropriately. Medical screening programs embedding vaccination within routine health assessments modestly increase immunization acceptance, particularly when combined with education and direct engagement (5, 26). This integrated approach may be more acceptable than standalone vaccination campaigns, as it frames immunization within holistic occupational health rather than isolated mandates.

### 4.5 Structural and policy implications

The finding that vaccination is infrequently mentioned within general preventive measures indicates limited cultural integration into operational safety frameworks—a critical gap, given that high vaccination coverage is considered essential for maintaining operational capability and preventing workforce disruption during outbreaks (9). International vaccination regulations for emergency services predominantly emphasize recommendations rather than mandates (10, 11, 36), creating implementation inconsistencies that disproportionately affect volunteer units that lack dedicated occupational health infrastructure.

Digital health documentation has received strong support contingent on privacy safeguards, suggesting readiness for modernized vaccination tracking systems that could address documentation deficits undermining current surveillance efforts (8). However, implementation must navigate complex data governance requirements and ensure voluntary participation to maintain institutional trust.

### 4.6 Limitations

This study has several limitations that should be considered when interpreting the findings. First, vaccination status, preventive behaviors, and hazard perceptions were self-reported and may therefore be subject to recall bias and social desirability bias. Second, participant recruitment relied partially on firefighters' networks and social media channels, potentially introducing self-selection and sampling bias. Volunteers with higher health engagement or stronger opinions about vaccination may have been more likely to participate. Third, this predominantly male sample, reflecting volunteer firefighter demographics in Germany, precludes a robust gender analysis. Cluster analysis, while revealing distinct behavioral profiles, requires validation in larger cohorts with standardized vaccination verification methods. The cross-sectional design of this study does not allow for the establishment of causality between risk perception, social integration, and vaccination behavior. Nevertheless, this mixed methods investigation provides rich contextual insights but may lack generalizability beyond the population sampled, especially in other countries with different organizational structures, training, and deployment requirements, or healthcare systems. Nonetheless, the identified pattern offers important insights into preventive health engagement within decentralized emergency response systems.

## 5 Conclusion

Volunteer firefighters demonstrate nuanced and context-specific infection risk awareness but show substantial gaps in vaccination knowledge, incomplete immunization documentation, and heterogeneous preventive behavior patterns shaped by professional background, institutional trust, and social norms. Effective interventions must address structural barriers, such as access, cost, documentation systems, and provide transparent and intuitive evidence-based communication; integrate vaccination into valued occupational health surveillance practices; and account for behavioral heterogeneity by adopting differentiated rather than uniform approaches. Strengthening vaccination coverage in decentralized emergency response systems represents both a public health priority and an operational resilience imperative that requires systematic policy attention and resource investment.

## Data Availability

The datasets used and/or analyzed in the current study are available from the corresponding author upon reasonable request.

## Supplementary material

Supplementary Appendix 1 Interview guideline.
