# Supplementary material for: Vaccination gaps in decentralized emergency response: understanding immunization barriers among volunteer firefighters. A cross-sectional mixed methods study
Source: Front Public Health. 2026 Apr 10;14:1803949. doi: 10.3389/fpubh.2026.1803949 (PMC13106354; doi:10.3389/fpubh.2026.1803949)
Supplement: Supplementary Appendix 1 — Interview guideline. [file Supplementary_file_1.pdf]

Projekt – Gefahrensensibilität und Impfkonzeptentwicklung in der Freiwilligen Feuerwehr
PD Dr. Franzen/ PD Dr. Mortensen / Brandt

Themen für das semistrukturiertes Interview (qualitative Datenerhebung) – Aktive der FF

1. Person und Organisation

1.1. Was machen Sie hauptberuflich?

1.2. Seit wann sind Sie Mitglied bei der Freiwilligen Feuerwehr?

1.3. Mit welcher Motivation sind Sie damals der Freiwilligen Feuerwehr beigetreten?

1.4. Engagieren Sie sich in weiteren Hilfsorganisationen?

1.5. Zu wie vielen Einsätzen werden Sie schätzungsweise pro Monat oder pro Jahr zur Hilfe gerufen und bei wie vielen von diesen treten Sie i.d.R. an?

1.6. Zu welcher Art von Einsatz wurden Sie oder ihre Kameraden und Kameradinnen in der Vergangenheit/in Ihrer Laufbahn hauptsächlich gerufen?

1.7. Erkennen Sie eine Veränderung hinsichtlich der Einsatzstellen? Gab es Einsätze in den letzten paar Jahren, die sie zu Beginn ihres Ehrenamtes nur selten oder gar nicht erlebt haben? Wenn ja, welche?

1.7.1. Waren Sie bereits Helfer bei Einsätzen bedingt durch klimatische Veränderungen (z.B Waldbrände oder Überschwemmungen) oder bei Einsätzen der Flüchtlingshilfe?

1.8. Erwarten Sie in Zukunft eine andere Art von Einsätzen? Wenn ja, welche und warum?

⇨ In Bezug auf Klimawandel / Kriege --> Flüchtlinge?)

2. Mögliche Gefahren, die von einer Einsatzstelle ausgehen

2.1. Welche Gefahren setzen Sie sich an einer Einsatzstelle im Allgemeinen aus?

2.2. Haben Sie in der Vergangenheit eigene Schäden oder Verletzungen in Einsätzen bzw. an Einsatzstellen erlitten?

2.2.1. Wie sind Sie damit umgegangen? (An wen haben Sie die Verletzung gemeldet? Von wem wurde die Verletzung behandelt? Wer übernahm die Kosten? Welche Maßnahmen haben Sie getroffen, um weitere Schäden/Verletzungen zu vermeiden?)

2.3. Welche möglichen Gefahren bestehen bei klimabedingten Einsätzen?

2.3.1. Starkregen / Überschwemmungen?

2.3.2. Hitze / Waldbrände?

2.4. Gibt es besondere Gefahren bei Arbeiten in der Flüchtlingshilfe?

2.5. Kennen Sie den Begriff *Infektionskrankheiten*? Wenn ja, können Sie diesen kurz erklären?

2.6. Welche Übertragungswege von Infektionskrankheiten kennen Sie?

2.7. Glauben Sie, dass Infektionskrankheiten eine Gefahr an Einsatzorten darstellen?

2.7.1. Welche Infektionskrankheiten insbesondere?

3. Präventive Maßnahmen

3.1. Welche allgemeinen Schutzmaßnahmen (z.B. PSA, Übungen) werden getroffen, um Verletzungen oder Schädigungen an Einsatzstellen zu vermeiden? (Auch in Bezug auf Infektionskrankheiten)

3.2. Ist Ihnen ihr aktueller Impfstatus bekannt?
- ⇨ Vor allem Tetanus, Hepatitis A und B?
- ⇨ Aber auch MMR(V), Polio, Diphterie, FSME?

3.3. Falls Sie sich für andere Hilfsorganisationen engagieren – spielt der Impfstatus oder die Beschäftigung mit dem Impfen eine Rolle?

3.4. Kennen Sie den Nutzen von Impfungen? (Eigenschutz, Herdenimmunität)

3.5. Welche Impfungen halten Sie für die Tätigkeit bei der Freiwilligen Feuerwehr für sinnvoll?

3.6. Welche Informationen wünschen Sie sich im Vorfeld zum Impfen zu erhalten?

3.7. Glauben Sie, dass ein Incentive die Impfbereitschaft erhöhen würden?

3.8. Ist Ihnen bewusst, dass bei fehlender Impfung ggf. eine fehlende Einsatzbereitschaft besteht und die Teilnahme an Einsätzen durch die Wehrführung verwehrt werden kann?

3.8.1. Würden Sie sich impfen lassen, wenn eine berufliche Indikation besteht?

3.9. Wären Sie bereit an einer arbeitsmedizinischen Vorsorgeuntersuchung teilzunehmen?

3.9.1. Welche Ansprüche bzw. Erwartungen hätten Sie an diese Untersuchungen?

3.9.1.1. Welche Untersuchungen sollen durchgeführt werden?

3.9.1.2. Über welche Themen würden Sie gerne aufgeklärt werden?

3.10. Wären Sie bereit eine Chipkarte oder einen Dienstausweis mit relevanten Gesundheitsinformationen bei sich zu tragen?
- ⇨ Relevante Gesundheitsinformationen = Blutgruppe, Organspender, Impfstatus, Medikamente, Allergien?
